# Systemic Lupus Erythematosus Presenting as Cardiac Tamponade and Pleural Effusion: A Case Report

**DOI:** 10.7759/cureus.52894

**Published:** 2024-01-25

**Authors:** Alhareth M Amro, Salah Deeb, Rama Rije, Nour Deeb, Yaman Y Qunaibi, Bajis Amr, Khaled Irzeqat, Baha Alhadad, Ahmad Emar

**Affiliations:** 1 College of Medicine, Al-Quds University, Hebron, PSE; 2 College of Medicine, Palestine Polytechnic University, Hebron, PSE; 3 Department of Cardiology, Al-Ahli Hospital, Hebron, PSE

**Keywords:** systemic lupus erythematosus, pericardial tamponade, pleural effusion, impending pericardial effusion, cardiac complications

## Abstract

Systemic lupus erythematosus (SLE) is a chronic inflammatory disease that can affect the heart, lungs, and other organs. We describe the case of a 36-year-old female patient who first presented with non-specific symptoms before receiving a diagnosis of SLE, along with initial evidence of pleural effusion and cardiac tamponade. Heart tamponade, which is characterized by fluid accumulation in the pericardial space, is an unusual but serious side effect of SLE. Pleural effusion, or an accumulation of fluid in the pleural cavity, is a typical hallmark of SLE; however, it rarely manifests as the disease's initial symptom. The early identification and diagnosis of these cardiovascular symptoms of SLE is critical for timely intervention and improved patient outcomes. This case report highlights the significance of considering SLE when performing a differential diagnosis for patients who have cardiovascular symptoms, particularly when pleural effusion and cardiac tamponade are present. To increase awareness and knowledge of these uncommon presentations of SLE, more investigations and comprehension of the underlying pathophysiology are required.

## Introduction

Systemic lupus erythematosus (SLE) is an inflammatory chronic condition defined as an increase in autoantibodies that may impact several body parts like the skin, joints, lungs, neurological system, serous membranes, and/or other body organs [1]. One of the most commonly affected organs in SLE is the heart. Pericardial effusion, pericarditis, Libman-Sacks endocarditis, myocarditis, and, most seriously, coronary heart disease (CHD) are all examples of cardiac involvement, leading to significant cardiac morbidity and mortality [2]. Pericardial effusion is the most common cardiac manifestation of SLE. The progression to developing cardiac tamponade is very rare, with an incidence between 1% and 3% [3].

The involvement of the heart as an early manifestation of SLE has been documented in the literature. Cardiac tamponade is a rare manifestation of SLE, and when it occurs as an initial clinical finding in SLE patients, it is even rarer [4]. Lupus pleuritis stands as the preeminent pulmonary manifestation of SLE. Pleural effusion alone may appear as an extremely rare initial sign of SLE [5].

In this case report, we describe a young female who presented with non-specific symptoms and was found to have a massive pericardial effusion and bilateral pleural effusions (mild in the right and moderate in the left pleural cavities) as the presenting clinical findings of SLE. This paper intends to elaborate on the pathophysiological underlying cardiac tamponade, delineate the infrequent occurrence of this presentation as a manifestation of SLE, and attempt to elevate awareness regarding SLE's potential presence within the spectrum of differential diagnoses for cardiac tamponade and pleural effusion within the context of relevant clinical scenarios.

## Case presentation

A 36-year-old, 10-day postpartum female presented with complaints of chest pain, fatigue, dyspnea, and fever over the past month. Her chest pain was on the left side, described as "stabbing," radiated into her left shoulder, and was related to increased exertion. It was not associated with palpitations. She had an unremarkable past medical or surgical history. During her stay in the hospital, the patient was febrile and became increasingly fatigued. One day later, she became diaphoretic and developed dizziness, and her dyspnea significantly worsened. She denied flu-like symptoms recently or intravenous (IV) abuse. She denied dental caries, hair loss, oral ulcers, a rash, joint pain, or chest trauma. There is no family history of autoimmune diseases. She doesn't drink alcohol or smoke cigarettes.

On physical examination, her heart rate was 108 beats per minute (bpm); other vital signs were normal except for the temperature which was measured at 36.3°C. She had pale skin and pale conjunctiva. Head and neck examinations were normal; she had no goiter or cervical lymphadenopathy, but it has been noticed that she has axillary lymphadenopathy. Her abdomen was soft and lax, but significant guarding at the site of her uterus was recognized. The respiratory examination determines the absence of breath sounds and dull percussion on the left lower hemithorax. On cardiovascular examination, cardiac auscultation revealed attenuated heart sounds, accompanied by a diffuse and inferiorly displaced apex beat. Jugular venous distension was clinically apparent. There were no manifestations of joint inflammation, deep vein thrombosis (DVT), peripheral edema, or clubbing. Neurological, psychological, and musculoskeletal exams were normal.

**Figure 1:**
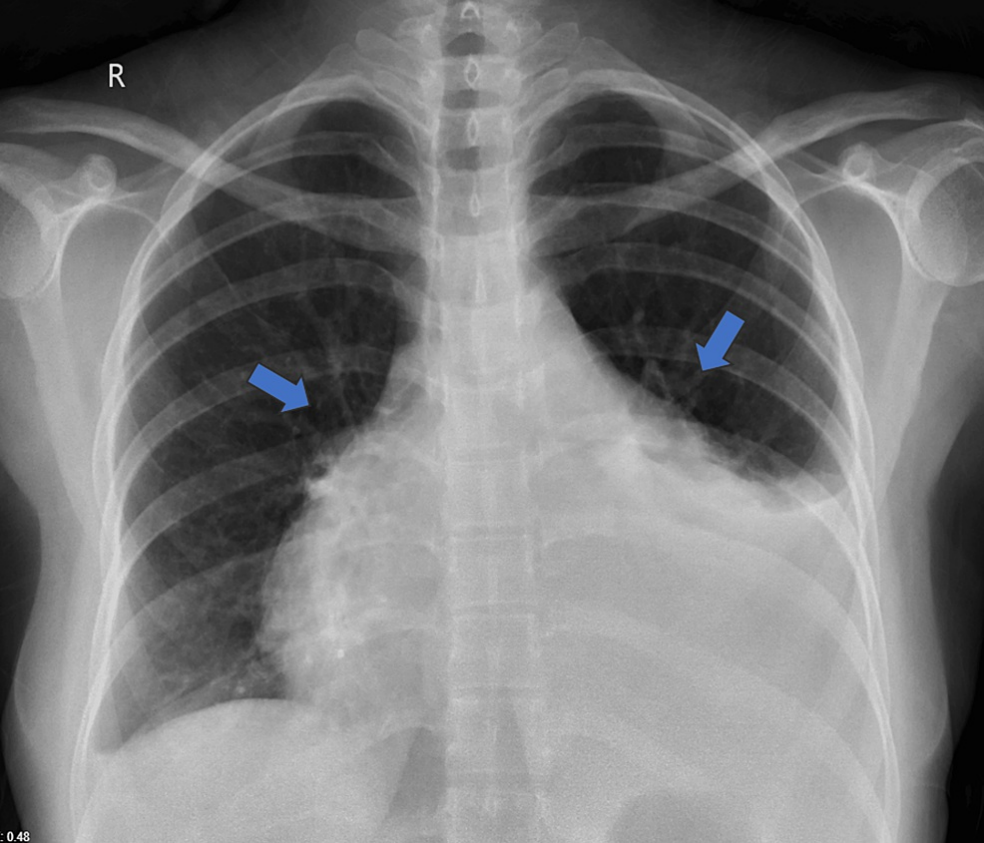
Chest X-ray shows an enlarged cardiac silhouette and moderate left pleural effusion. Blue arrows show cardiac silhouette sign.

**Figure 2:**
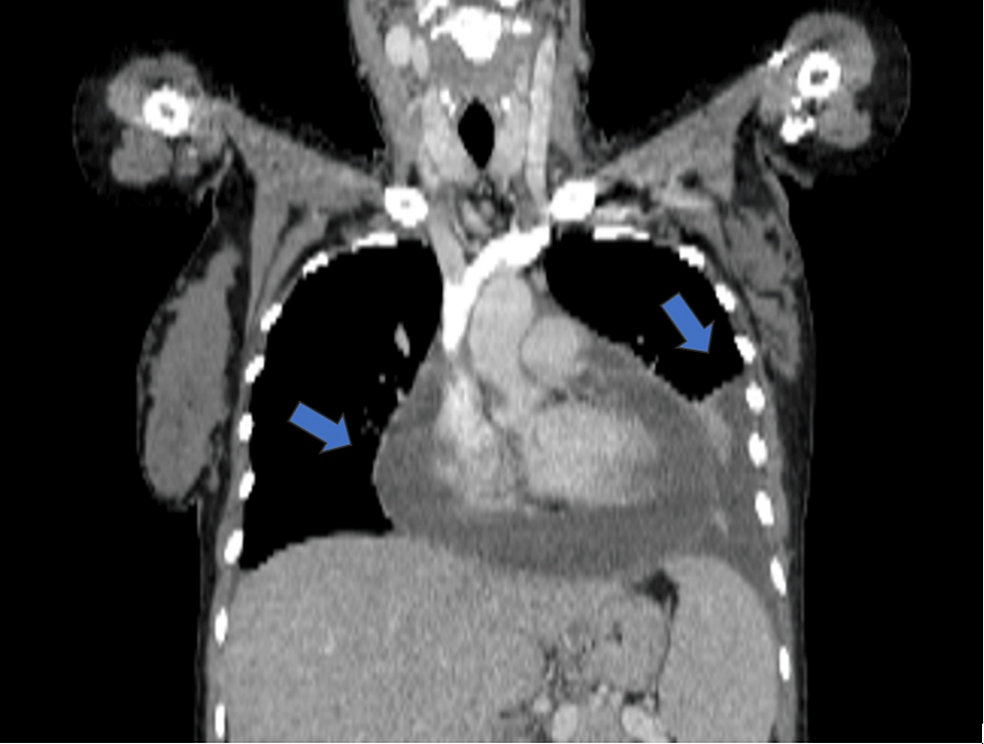
CT scan shows pericardial effusion and left pleural effusion. Blue arrows show pericardial effusion with left pleural effusion.

Lab and imaging studies were done. Electrocardiography showed sinus tachycardia. The chest X-ray shows an enlarged cardiac silhouette with a moderate left-sided pleural effusion (Figure 1). Transthoracic echocardiography (TEE) revealed features of moderate-to-severe cardiac tamponade circumferentially pericardial effusion inferoposteriorly, with mobile fibrinous bands of the intrapericardial and exudative pleural effusion. The left and right ventricles were of normal size and contracted well. There were no regional wall motion abnormalities (RWMA). Both atria were normal. Mild tricuspid regurgitation was detected. The inferior vena cava was dilated, not collapsed. Chest CT showed a pericardial effusion and a pleural effusion, as shown in Figure 2.

Laboratory investigations revealed normochromic normocytic anemia, no increased schistocytes, and no nucleated red blood cells (NRBCs). Other laboratory values are shown in Table 1. Stool occult blood was positive. Iron studies for anemia and upper and lower endoscopy were done to rule out malignancy or connective tissue disease. A manual breast exam and gynecological consultation were done. Breast ultrasound showed axillary lymph node enlargement. Whole-body CT was refused by the patient. The patient's serum tested strongly positive for anti-Sjögren's syndrome A (SS-A) and anti-Sjögren's syndrome B (SS-B) antibodies and was also positive for anti-double-stranded DNA (anti-dsDNA) antibodies. Additional tests revealed an elevation in serum complement levels and cancer antigen 125 (CA-125), but other malignant markers are negative. Pleural fluid cytology showed cells of an inflammatory response with no evidence of malignancy.

**Table 1:** Patient's laboratory values Hb: hemoglobin; HCT: hematocrit; WBC: white blood cells; INR: international normalized ratio; MCV: mean corpuscular volume; LDH: lactate dehydrogenase; C3: complement 3; c4: complement 4; ESR: erythrocyte sedimentation rate; CRP: C-reactive protein

Blood work	Patient test result	Normal range	Interpretation of the test
Hb	7.5 mg/dL	13.5-17.5 mg/dL	Decreased
HCT	22.7%	36-48%	Decreased
MCV	78.3 fl	80-100 fl	Decreased
WBC	4,200/mm^3^	4,500-11,000/mm^3^	Mild decreased
Lymphocytes	15.3%	25-45%	Decreased
Neutrophils	78.5%	45-65%	Elevated
Platelet	226,000/mm^3^	150,000-450,000/mm^3^	Normal
INR	1.5	0.8-1.1	Elevated
Iron	14 ug/dL	37-145 ug/dL	Decreased
Serum LDH	220	250-450	Decreased
C3	243 mg/dL	75-175 mg/dL	Elevated
C4	52 mg/dL	15-45 mg/dL	Elevated
Creatinine	0.8 mg/dL	0.7-1.3 mg/dL	Normal
Alkaline phosphatase	147 IU/L	44-147 IU/L	Normal
Calcium	8.5 mg/dL	8.5-10.5	Decreased
Triglyceride	214 mg/dL	Less than 150 mg/dL	Elevated
ESR	150 mm/hour	0-20 mm/hour	Elevated
CRP	84 mg/L	0-6 mg/L	Elevated
Serum sodium	138 mmol/L	133-146 mmol/L	Normal
Serum potassium	2.4 mmol/L	3.5-5.5 mmol/L	Decreased
Serum chloride	104 mmol/L	95-110 mmol/L	Normal

Her potassium was decreased, so she was given potassium chloride (KCL) orally and in a slow IV infusion. The iron profile showed iron deficiency anemia. Coombs test, serologic tests for hepatitis C virus (HCV), hepatitis B surface antigen (HBsAg), and human immunodeficiency virus (HIV), and a tuberculin skin test were negative. Cardiac biomarkers were all normal (troponin was negative).

The diagnosis of SLE was ascertained through affirmative clinical and immunologic parameters. The patient met the criteria established by the Systemic Lupus International Collaborating Clinics (SLICC), fulfilling four of the 17 specified criteria for SLE classification. These included serositis, diminished serum complement levels, positive serum antinuclear antibodies (ANA), affirmative anti-dsDNA antibodies, as well as positive anti-SSA and anti-SSB antibodies. Additional support for the diagnosis was derived from the patient's reported history of fatigue, generalized lymphadenopathy, and an elevated erythrocyte sedimentation rate (ESR). Subsequently, corticosteroid therapy was initiated, colchicine 0.5 mg 1×2 per os (PO), Trufen 600 mg (Arogga, Dhaka, Bangladesh) 1×3 PO for two weeks, Nexium 40 mg (Pfizer, New York, New York, United States) 1×1 PO, Rocephin 2 g (Roche, Basel, Switzerland) 1×1 IV, Slow-K 600 mg (Geryon, Speke, England) 2×3, and folic acid 5 mg 1×1. Repeated echocardiography tests every day showed very good progressive improvement of the effusion which became milder every day until day 5 of admission. At that time, echocardiography showed mild circumferential pericardial effusion with very mild left-sided pleural effusion. At the follow-up visit four months after discharge, she maintained a favorable clinical course and was enrolled in the clinic for the purpose of long-term management of SLE.

## Discussion

Our patient presented with symptoms of left-sided chest pain radiating from her shoulder, fatigue, fever, and dyspnea on exertion. Examination showed tachycardia, tachypnea, hypotension, jugular venous pressure elevation, and muffled heart sounds, leading to a cardiac tamponade diagnosis. A moderate left-sided pleural effusion was found on a chest CT. Electrocardiography revealed sinus tachycardia with electrical alternans. Electrical alternans, in which there is an alteration in the amplitudes of the QRS complexes with every other beat, are an unusual finding in cases of cardiac tamponade, with reported sensitivities of only 8-21% [6]. However, it is highly indicative of pericardial effusion, with a specificity of 89% and a positive predictive value of 82% [6]. These features are highly suggestive of pericardial effusion and tamponade. Chest radiography demonstrated an enlarged cardiac silhouette with an infiltrate in the left pleural cavity, an integration recorded to have a positive predictive value for pericardial effusion. The initial presentation of SLE in our patient was a cardiac tamponade. Also, the laboratory values showed high anti-nuclear antibody titers, anti-dsDNA antibodies, and anti-SSA antibodies. A conventional physical examination finding, such as Beck's triad, is an independable pericardial effusion or tamponade diagnosis [6]. Our patient had normochromic, normocytic anemia.

The most common cardiac manifestation of SLE is pericardial effusion, which occurs in up to 50% of patients somewhere in the course of their illness. It is common and potentially life-threatening, but it can be treated. It is usually mild and unusually seen early in the course of the disease; in rare cases, it leads to cardiac tamponade. Also, the pleural effusion can be due to SLE polyserositis [7,8]. In some cases, the pericardial effusion can be due to renal failure rather than lupus serositis [9].

A workup for SLE is necessary when the underlying cause of cardiac tamponade cannot be readily explained by any disease process, even though it is incredibly rare [6,10]. Cardiac tamponade in SLE can occur during the course of the disease or as its initial presentation. It is a medical emergency that needs early diagnosis and treatment to reduce morbidity and mortality [7]. It must be well known that cardiac tamponade in SLE can be from both circumferential and loculated effusions; the latter gives an atypical picture and is called regional tamponade. The progression to tamponade in these patients is affected by a group of factors that can vary from patient to patient, which complicates the identification of tamponade predictors [8]. A significant predominance of females was noted [8,6]. If an adolescent female presents with hemodynamic compromise due to cardiac tamponade, wSLE should be considered as a possibility in the differential diagnosis, especially if features and findings are suggestive of a rheumatic process [10]. Knowing that tamponade can occur at any point in their disease, this life-threatening diagnosis should be considered in patients who have unexplained venous congestion [6].

Pericardial involvement is also somehow common in other connective tissue diseases like systemic sclerosis (>60%), mixed connective tissue disease (10-30%), rheumatoid arthritis (10-30%), Sjögren's syndrome (<30%), adult Still's disease (<30%), polymyositis, and dermatomyositis (<10%). It occurs rarely in sarcoidosis [6].

The size of the pericardial effusion is an important determinant in the development of tamponade. However, in patients with lupus, tamponade can occur in moderate- and even small-sized effusions [11]. This size is a good indicator of tamponade, but there is a wide variability in the volumes of effusion that are drained on pericardiocentesis. This ensures that although patients are more likely to decompensate with these effusions, because of the subacute accumulation in SLE, the critical volume at which tamponade will develop differs from case to case [8]. However, echocardiography can detect small and silent pericardial effusions [6].

The diagnosis of tamponade is made when there is evidence of right-sided cardiac chamber collapse, dyspnea, hypotension, tachycardia, and elevated jugular pressure, along with pericardial effusion or pulsus paradoxus. The majority of studies have reported on low serum C4 levels below normal, and 50% or more of tamponade cases showed this finding. However, distinct case reports of SLE patients who suffered from tamponade had normal levels of serum C4. Generally, the clinical exacerbations might be able to be predicted by serial monitoring of C4 levels in serum. Another study was done to see if low C3 and C4 levels corresponded to a flare in SLE. A relationship between low C4 and SLE flare was unable to be established [12].

A low level of serum complement C4 signifies the advancement of cardiac tamponade in patients with SLE [6]. Our patient had high levels of complement C4. Cardiopulmonary features of lupus like myocarditis and myositis are associated with anti-ribonucleoprotein (RNP) antibodies and engage features like myositis and Raynaud's phenomenon. The mitral valve was most commonly involved [9].

Echocardiography should be done, if feasible, to grade and localize the pericardial effusion and reveal pericardial thickening and the presence of intrapericardial adhesions, in addition to being a standard for confirming the diagnosis [6]. In our case, echocardiography obviously visualized a great circumferential pericardial effusion with distinctive signs of tamponade and a left-sided pleural effusion. Within the pericardial effusion, there were partially attached dynamic fibrinous bands traversing the visceral and parietal layers of the pericardium. The other rare cases of SLE that present with pericardial effusion increase the indication of a pericardiocentesis procedure [6]. The pericardial fluid is mainly described as hemorrhagic in appearance. Other markers to further support the diagnosis are antinuclear antibodies, the presence of immune complexes, and complement levels. Its protein concentration varies, being high in exudates and low in transudates. Glucose levels are within the normal range [7].

There is disagreement over C-reactive protein (CRP)'s role in the diagnosis of SLE activity. Up to a level of 50 mg/L, a recent study demonstrated a logical correlation with illness activity; readings over this threshold were more suggestive of a linked infection. It has been shown that the complement has a strong correlation with lupus activity. A lupus nephritis flare and a hematological flare are significantly correlated with a decline in C3 and C4, but no concomitant flares in other organ systems are observed [9].

Recent systematic reviews and meta-analyses concluded that anti-nucleosome antibodies have equal specificity but a higher sensitivity and prognostic value than anti-dsDNA antibodies when diagnosing SLE. There is an importance of concomitant pleuritis, anti-nucleosome antibodies, and the size of pericardial effusion in predicting the development of tamponade. We believe that immunosuppression by using methylprednisolone and IV cyclophosphamide is critical, especially to decrease the risk of reaccumulating fluid in the cavity and decrease the indication of pericardiocentesis procedure [11].

Previous reports have shown that anti-RNP antibodies are associated with pulmonary disease, specifically pleuritis, in SLE and in other connective tissue diseases. In the serologic profile, anti-Sm antibodies are reported as they are linked with an increased risk of central nervous system (CNS) disease, renal disease, and mortality in SLE. To explain this serologic observation, we depend on potential pathophysiologic mechanisms. Anti-RNP antibodies can play a role in the process of tissue injury and inflammation through the upregulation of inflammatory cytokine production and adhesion molecules at the level of endothelial cells (endothelial leukocyte adhesion molecule-1 and intercellular adhesion molecule-1), which is done through the expression of MHC class II on endothelial cells, and also via the interaction with Toll-like receptors [13].

Myocardial infarction due to coronary arteritis may be a significant cause of death in young adults with SLE [11]. The exact cause of it is probably multifactorial, involving immune myocarditis, small vessel vasculitis, and hypertension due to steroids [9].

Concomitant seropositivity for RNP and Sm antibodies, in addition to greater cumulative organ damage, a longer disease period, and the onset of disease at a younger age, were each associated with an elevated risk for prevalent lupus pleuritis. Constructing factors linked to pleuritis provides valuable information. We suggest that seropositivity for both RNP and Sm antibodies is linked with a higher likelihood of developing pleuritis. Therefore, we need to recognize the clinical relevance of pleuritis beyond its serologic and clinical correlates, which may not be possible in this cross-sectional study. Previous reports demonstrated a link between short-term mortality and pleuritis. Identifying SLE patients at risk for pleuritis and its consequences has been hampered in the past by a lack of predictors of pleuritis [13].

Using the SLICC criteria, our patient was diagnosed with SLE. However, our patient was unfit to satisfy four of the 11 American College of Radiology (ACR) criteria for classifying SLE. As observed in this case report, low complement levels have not been reported. The SLICC criteria have introduced anti-dsDNA antibodies, anti-Sm antibodies, and antiphospholipid antibodies to contribute to the diagnosis. By permitting a higher weighting of immunologic criteria, the use of the SLICC criteria could be more sensitive to diagnose such cases, possibly leading to an earlier diagnosis and treatment [6].

In treating known cardiac tamponade, efforts to withdraw the pericardial fluid by pericardiocentesis are the treatment of choice [7]. The echocardiography-guided pericardiocentesis has been associated with lower morbidity and mortality rates, compared to surgical pericardiectomy or placement of a pericardial drain, which are less commonly used. Medical management involves anti-inflammatory medications like nonsteroidal anti-inflammatory drugs (NSAIDs) and high-dose corticosteroids to reduce the frequency of pericardiocentesis and antimalarials [6]. Pericardiocentesis was not done for this patient. She became well, and her symptoms regressed with the medical treatment as mentioned in the case presentation.

Follow-up is mandatory to exclude recurrent pericardial thickening or effusions. In our case, during the admission, ESR and CRP levels were 150 mm/hour and 84 mg/L, respectively. ESR elevation commonly occurs during a flare, but CRP is usually normal or slightly elevated. Serositis is an SLE manifestation that increases both CRP and ESR levels [6].

It is crucial to include SLE in the differential diagnosis of patients who present with cardiovascular symptoms because cardiac tamponade and pleural effusion are uncommon early presentations of SLE. For prompt intervention and improved patient outcomes, early detection and diagnosis are essential. 

## Conclusions

The rare incidence of cardiac tamponade and pleural effusion as initial manifestations of SLE is explained by this case report. It highlights the difficulties in obtaining an early diagnosis and the significance of taking SLE into account when making a differential diagnosis for patients with cardiovascular symptoms. Raising awareness of these unusual appearances has the potential to save lives and improve patient outcomes. To determine the best management approaches and to gain a deeper understanding of the mechanisms underlying various manifestations, more research is necessary.
